# Applying systems thinking in youth-centred participatory action research for health promotion in an underserved neighbourhood

**DOI:** 10.3389/fpubh.2024.1272663

**Published:** 2024-06-03

**Authors:** Helga Emke, Teatske Altenburg, Coosje Dijkstra, Angie Luna Pinzon, Karien Stronks, Wilma Waterlander, Stef Kremers, Mai Chinapaw

**Affiliations:** ^1^Department of Health Sciences, Faculty of Science, Vrije Universiteit Amsterdam, Amsterdam, Netherlands; ^2^Amsterdam Public Health Research Institute, Health Behaviour and Chronic Diseases and Methodology, Amsterdam, Netherlands; ^3^Department of Public and Occupational Health, Amsterdam University Medical Centre, Vrije Universiteit Amsterdam, Amsterdam, Netherlands; ^4^Department of Public and Occupational Health, Amsterdam University Medical Centre, University of Amsterdam, Maastricht, Netherlands; ^5^Department of Health Promotion, NUTRIM School of Nutrition and Translational Research in Metabolism, Maastricht University Medical Centre, Maastricht, Netherlands

**Keywords:** participatory action research, systems thinking, adolescents, obesity, overweight

## Abstract

**Purpose:**

Childhood overweight is considered a complex problem influenced by a range of factors, including energy balance-related behaviours (EBRBs) and interacting drivers of these behaviours. There is growing support that applying a systems approach is required to tackle complex problems resulting in actions that attempt to change the system’s dynamics. Additionally, a participatory approach is advocated to include the lived experience of the population of interest both in the understanding of the system as well as the development, implementation and evaluation of relevant actions. We therefore combined Intervention Mapping, Participatory Action Research (PAR) and system dynamics in the development, implementation and evaluation of actions contributing to healthy EBRBs together with adolescents.

**Methods:**

Four PAR groups comprising of 6–8 adolescent co-researchers (10–14 years) and 1–2 adult facilitators met weekly during 3–4 years. The structured Intervention Mapping protocol guided the process of the systematic development, implementation and evaluation of actions. System dynamics tools were included for the creation of Causal Loop Diagrams and development of systemic actions.

**Results:**

Our approach comprised six steps that were executed by the PAR groups: (1) build Causal Loop Diagrams for each EBRB through peer research and identify overarching mechanisms, (2) determine leverage points using the Intervention Level Framework, (3) develop action ideas, (4) develop detailed actions including an implementation plan, (5) implement and, (6) evaluate the actions. PAR ensured that the actions fitted the lived experience of the adolescents, whilst system dynamics promoted actions at different levels of the system. The Intervention Mapping protocol ensured that the actions were theory-based. The main challenge involved integrating system dynamics within our practise in cooperation with adolescent co-researchers.

**Conclusion:**

We experienced that combining Intervention Mapping, PAR and system dynamics worked well in developing, implementing and evaluating actions that target different levels of the system that drive adolescents’ EBRBs. This study serves as an example to other studies aimed at developing, implementing and evaluating actions using a participatory and systems approach.

## 1 Introduction

Overweight and obesity are considered complex problems that emerge from the interplay between interacting factors ranging from individual characteristics to influences from the obesogenic physical, economic, policy, and sociocultural environment (1, 2). This web of factors is referred to as a complex system. Relevant factors in this web underlying overweight and obesity are elements relating to energy balance-related behaviours (EBRBs), including physical activity, screen use, sleep, and dietary behaviour, and the underlying drivers of these behaviours, which intricately interact and impact each other (3–5). The obesogenic environment is an important driver of these individual behaviours, overweight is therefore a result of people reacting normally to this particular environment (6, 7). An example is the extensive availability and marketing of cheap and energy-dense foods, partly caused by the lobbying forces of powerful food and beverage manufacturers (6, 7). Another example, is that urban outdoor environments are unattractive for active play–especially for adolescents (8).

Current interventions to improve the EBRBs are insufficient. Globally, the prevalence of children and adolescent overweight and obesity increased drastically from 4% in 1975 to over 18% in 2016 (9). In the Netherlands between 2018 and 2022, amongst 4–12-year-olds the prevalence of overweight remained steady at 12%, whilst for 12–16-year-olds, it rose from 12 to 14% (10). Amidst the COVID-19 pandemic, the prevalence of adolescents with overweight peaked at 16% amongst 4–12-year-olds in 2021 and 19% amongst 12–16-year-olds in 2020 (10). Particularly concerning is the higher prevalence amongst children and adolescents from families with a lower socioeconomic position (11). The widening health inequalities between social groups (11), suggest that existing interventions have either maintained or increased health inequalities (12). Families from a lower socioeconomic position often live in underserved neighbourhoods, which have less opportunities for physical activity and a substantially greater number of fast-food outlets (13, 14). Furthermore, families from a lower socioeconomic position are less likely to participate in interventions (15, 16), which could partly be because current interventions insufficiently match their needs and preferences.

Systems thinking is a promising approach to tackle complex problems, by providing a better understanding of the dynamics of the complex system that shape the EBRBs of adolescents (8), and subsequently supporting the development of actions that attempt to change the system’s dynamics by targeting underlying causes of the problem (6).Within systems thinking complex problems are explored and tackled by looking at the whole system consisting of an interconnected set of elements and feedback loops, instead of separate elements (17). For example, screen use leads to more unhealthy snacking, less physical activity, and lower sleep quality in adolescents (8). Lower sleep quality, in turn, leads to more snacking and less physical activity. This highlights the importance of tackling multiple EBRBs at once to tackle overweight and obesity amongst adolescents (8). One particular approach within systems thinking is participatory system dynamics, in which the population of interest is involved in the process of understanding and changing the system (18). Together, a shared understanding is created of both the dynamic nature of and interconnection between different elements of the system by developing a causal map of the system (19). Previous research utilising system dynamics with adolescents generated novel insights into certain drivers of adolescent obesity that were not well documented in existing research and policy, including the key influence of social media and mental health (20). Showing the importance of conducting a system dynamics approach whilst including adolescents’ perspectives. This is in line with the interpretative epistemology perspective, which acknowledges that the complex nature of the system can best be understood by developing knowledge together with academic researchers and the population(s) of interest (21).

Participatory Action Research (PAR) also aligns with the interpretative epistemological perspective, where academic researchers seek to understand a specific context through perceived knowledge (22, 23). PAR is promising to better understand and improve the system that shapes adolescents’ EBRBs from the perspective of adolescents themselves. PAR is characterised by collaboration and shared decision-making with the population of interest throughout the research process, with the aim to improve health and reduce health inequalities (24). Through PAR adolescents get the opportunity to be involved as co-researchers, which in turn provides them with the knowledge, skills and abilities that are required to conduct research on both their own particular context and that of their peers (24, 25). Participating in PAR has been found to improve adolescents’ individual development, empowerment, and critical awareness of societal issues (26), which enables them to reflect on problems they experience and take action. A previous PAR project with adolescents aged 9–12 showed that the adolescent co-researchers had an increased awareness of EBRBs, as well as improved confidence, critical awareness, leadership and collaboration skills (27). The community partners involved in this particular PAR project valued the contribution of the adolescents and the actions they developed (27).

With both PAR and system dynamics there is emphasis on the interaction of context and interventions. As described by the context and implementation of complex interventions framework, the setting (i.e., the specific physical location) interacts with the context and the implementation of the intervention (28). Similarly, the setting and context influence the development of interventions by the population of interest, based on their insights of the system and their needs and interests. Consequently, developed interventions may vary between different settings and contexts.

When developing actions to improve adolescent EBRBs it can be challenging to align ideas from the population of interest with theory and/or empirical findings from the literature, combining the interpretative perspective and the post-positivist perspective (29). The post-positivist perspective strives to provide the most objective evidence and often includes quantitative data (19, 21). The Intervention Mapping (IM) protocol is a planning framework used for the development, implementation and evaluation of theory-and evidence-based health promotion programmes. This systematic and stepwise protocol can be used to structure the development and implementation of actions, taking into account both the perspectives of the population of interest as well as the current evidence-base from literature (29).

Combining the IM, system dynamics and PAR approaches could prove critically important for developing interventions that tackle the complex problem of adolescent overweight. In this combination, IM provides a systematic and stepwise framework for developing theory- and evidence-based interventions, whilst system dynamics techniques can provide an understanding of the complexity of the system that influences adolescents’ EBRBs. PAR provides guidance on how to effectively involve the population of interest in such a way that ensures that the interventions match their specific needs and interests. However, no studies exist that combine these approaches effectively. Therefore, this paper describes how we combined IM, PAR and system dynamics in the development, implementation and evaluation of actions contributing to healthy EBRBs together with adolescents.

## 2 Methods

**Figure 1:**
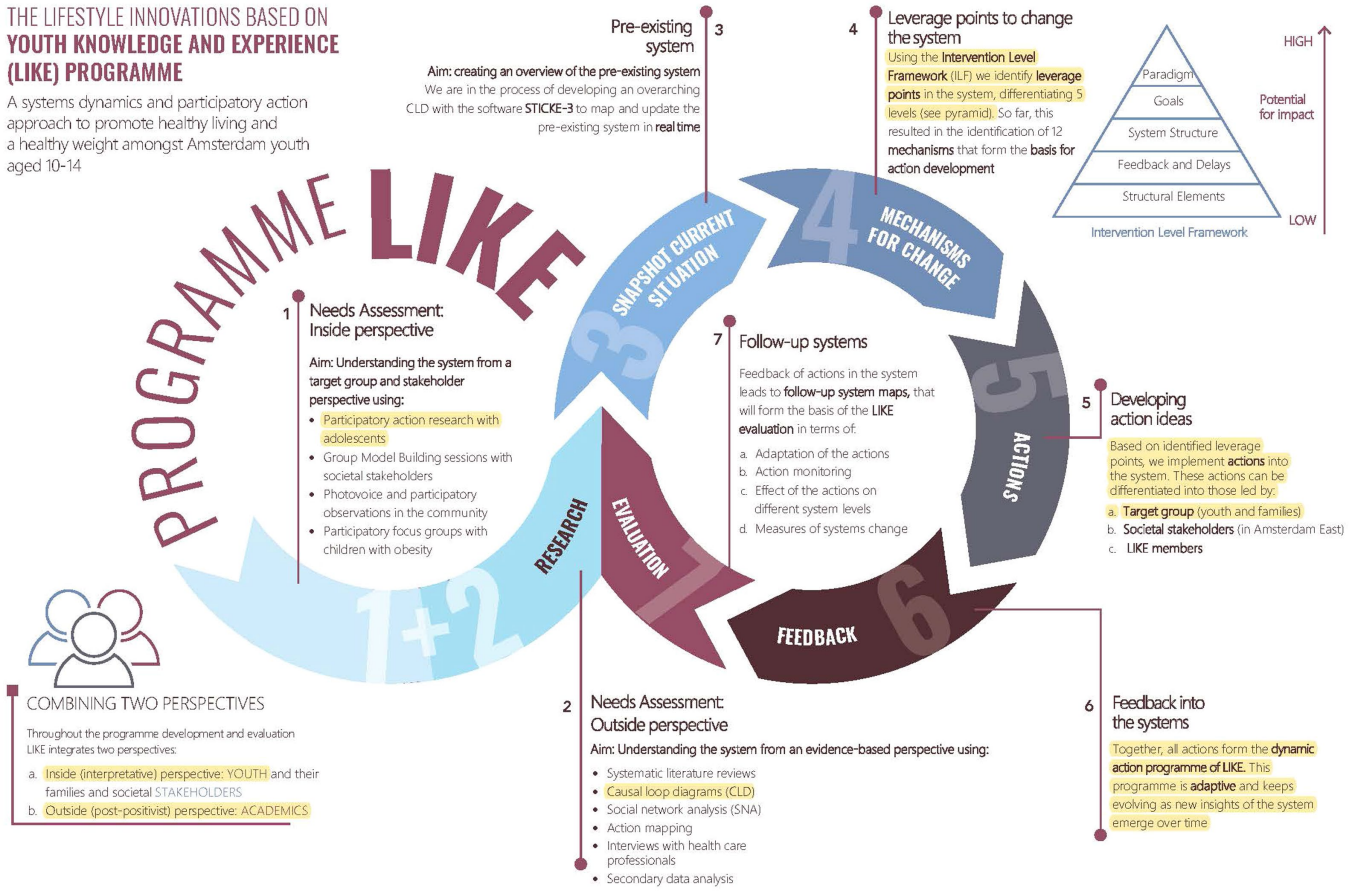
Overview of LIKE programme, with highlighted sections described in the present paper. Reprinted from Luna Pinzon et al. (19). Reprinted with permission from the author.

This study is part of the Lifestyle Innovations Based on Youth Knowledge and Experience (LIKE) programme that aims to tackle the complex problem of overweight and obesity amongst adolescents aged 10–14 living in an underserved neighbourhood in Amsterdam East, the Netherlands (23). LIKE is part of the Amsterdam Healthy Weight Programme which is a municipality-led systems approach that has been running since 2013 with the aim of preventing children in Amsterdam from becoming overweight (30). Figure 1 presents an overview of the LIKE programme (19). The highlighted sections in Figure 1 refer to the parts described in the present paper. Although the steps are presented in a linear way, in reality the process is more iterative (Figure 1). The Medical Ethical Committee of the VU University Medical Centre approved of the study protocol (2018.234).

### 2.1 Procedures and participants

The current study took place in three communities in Amsterdam East that were defined as high-priority neighbourhoods by the Amsterdam Healthy Weight Programme, based on the relatively high percentage of adolescents with overweight (ranging from 24 to 31% of 10 year olds) (31, 32). These communities had a relatively high rate of people with a non-western background (34–44%), and primary school children from families with a lower education (ranging from 26 to 28%) and from long-term poor households (ranging from 15 to 18%) (32).

How the participants were recruited is described in more detail in our needs assessment paper (33). Simply put, the participants were adolescents aged 10–14, attending primary or secondary schools that were located in one of the communities of interest with at least 50% of their students living in Amsterdam East. Four primary schools and five secondary schools, met the inclusion criteria and were invited to take part in the study by employees of the Amsterdam Healthy Weight Programme, who had already established contact with the schools. Two primary schools and two secondary schools never replied, whilst one secondary school declined to participate. Thus, a total of two primary schools and two secondary schools agreed to participate. Within the participating schools, adolescents from the final two grades of primary schools and the first two grades of secondary schools were invited to join the PAR groups. As most adolescents participated for one school year, recruitment of new PAR members was repeated yearly in each school. The primary school adolescents participated after school hours on a voluntary basis, whereas the secondary school students participated during school hours by registering for an elective course. One PAR group in each school was formed, comprising of 6–8 adolescent co-researchers and an academic researcher who facilitated the sessions.

### 2.2 Intervention mapping, system dynamics, and participatory action research

In this study we have combined the IM, system dynamics and PAR approaches, in which the PAR approach was leading. We will first explain the approaches separately before describing the combined approach per IM step. In the results section, we describe how these steps were executed in real life including more detail.

#### 2.2.1 Intervention mapping

The IM protocol comprises six steps structuring the development, implementation and evaluation of theory- and evidence-based interventions (29). The first step in the traditional IM protocol includes the assessment of the health problem, the needs of the population of interest and the specification of the intervention goals for better health and quality of life. The second step specifies who and what needs to be changed to improve the problem. In this step, it is advised to make matrices for each actor and topic along with change objectives, which state precisely what needs to be changed for each of the determinants in order to achieve the specific objectives for the intervention. In the third step, ideas for interventions are generated, followed by the identification of theoretical methods and practical applications for identifying behaviour change methods that are potentially effective in changing the behavioural determinants. This step culminates in the design of the intervention. During the fourth step, the intervention is made more specific by consulting both the participants and the implementers. The intervention is subsequently refined, the materials are produced, and the intervention is then pilot tested and produced. In the fifth step, potential stakeholders that can adopt the intervention are identified and a plan for adoption and implementation is developed. The sixth and final step concerns the development of the evaluation plan. This step includes the effect and process evaluation (29).

#### 2.2.2 System dynamics

We used two methods from the system dynamics toolbox, namely the development of Causal Loop Diagrams (CLDs) and the Intervention Level Framework (ILF) (34, 35). Within system dynamics, CLDs are used to visualise the dynamic complexity of the targeted problem by showing how determinants interact with each other through linkages and feedback loops (36). These feedback loops and connections between determinants can be used to describe overarching mechanisms in the system, for example the food environment or the responsibility of parents for their children’s health behaviour (8). The understanding obtained from these CLDs can then serve as input for action development.

In order to promote the identification of leverage points and development of actions on multiple levels of the system, the ILF was used. The ILF is adapted from Meadows, who identified 12 places to intervene in complex systems (17). Johnston et al. (34) then summarised this into five system levels: paradigm, goals, system structure, feedback and delays and structural elements. The paradigm is the highest systems level and consists of a system’s deepest held beliefs, which are difficult to change (e.g., active outdoor play is considered a routine behaviour amongst adolescents). The goals are targets that conform to a system’s paradigm and they need to be achieved in order for a paradigm shift to occur (e.g., the system serves the needs of adolescents). The system’s structure refers to the interconnections between the elements of the system and its subsystems, with actions on this level having the capacity to change the structure of the system (e.g., urban design planners and youth representatives collaborate on a regular basis). Feedback and delays allow the system to regulate itself through reinforcing or balancing feedback loops (e.g., outdoor facilities are attractive, and adolescents therefore make more use of them, in turn, making it more attractive for other adolescents). Finally, the level structural elements contains multiple factors such as subsystems, actors and physical elements, and is the easiest level to change (e.g., secondary schools encourage adolescents to use outdoor facilities) (34).

#### 2.2.3 Participatory action research

In this study the PAR approach was applied by collaborating with adolescents as co-researchers throughout the duration of the study, from needs assessment, data collection, data analysis and development of actions, up until the implementation and evaluation of the actions aimed toward improving adolescents’ EBRBs. Doing so ensured that adolescents’ perspectives were drawn upon in each and every aspect of the research process. Our PAR approach was guided by self-determination theory, which posits that people require three elemental needs to experience positive well-being during the sessions: (1) autonomy, (2) competence and (3) relatedness (37). When these needs are fulfilled, it helps to promote self-motivation and good mental health (37). When conducting PAR with adolescents as co-researchers we strove to meet each of these needs within the PAR sessions. More specifically, we sought to improve the co-researchers’ autonomy by helping them improve both their own lives and that of their peers (24). The current project was researcher-initiated, implying that preceding the formation of PAR groups, academic researchers determined that the problem of overweight and specific EBRBs were the focus of the project. To the co-researchers this was communicated as improving a healthy lifestyle of adolescents. Once the PAR groups were formed the facilitators promoted the adolescents’ autonomy by letting them in their capacity as co-researchers influence the research process, in order to establish a collectively owned research process (38). For example, by letting them choose their research method and the goal of their action. By providing the co-researchers with knowledge, skills and abilities, they gained competence in conducting research and related skills (25). In the present study, the co-researchers were trained through capacity building, which involved them learning basic research principles and methods that enabled them to conduct peer research, employ various system dynamics methods, and help them to develop theory-based actions, along with other important daily skills such as organisational skills. This also included ethical considerations and how to protect the privacy of their peers. To improve the co-researchers’ relatedness, we focused in the first sessions on creating a safe environment and sense of team spirit by getting to know each other through informal chatting and via specific introductory, teambuilding and group forming games. Maintaining this safe environment and team spirit required continual effort during the PAR groups across all the steps in the research process. We also began each PAR session with a check-in exercise, which consisted of a game or a short informal chat, aimed toward maintaining the safe environment and team spirit, as well as helping them to get settled in the session.

### 2.3 Combining PAR, system dynamics and IM

#### 2.3.1 Step 1: mapping the system

This step involved conducting a participatory needs assessment, which was subdivided into two steps: (1a) gaining insight into the perspectives of adolescents regarding factors influencing EBRBs (physical activity, screen use, sleep behaviour and dietary behaviour), and (1b) gaining insight into how these perceived factors are connected with one another.

##### 2.3.1.1 Step 1a: gaining insight into energy balance-related behaviours

To prepare the conducting of peer research amongst their schoolmates, the co-researchers first gained insight into their own EBRBs. We anticipated that this would help the PAR groups to formulate research questions for their peer research. Each PAR group focused on one or two EBRBs, including physical activity, screen use, sleep behaviour and dietary behaviour. The facilitators explained the following methods, from which the co-researchers could then select their preference: making a timeline of their day, a safari through the neighbourhood, searching for information on the internet and presenting reasons for unhealthy behaviour to each other. After gaining insight into the EBRBs, the co-researchers were then taught research skills by an academic researcher (introduced as ‘expert on research methods’). Thereafter, the PAR groups conducted peer research using various research methods and analysed the data. The facilitator assisted the co-researchers in interpreting the data, specifically in terms of identifying factors related to unhealthy EBRBs as well as the interactions between these factors and behaviours. The co-researchers then presented their data to their classmates or school board via, for example, a poster presentation in order to increase awareness of the project. The rationale for this was that we felt the project would have a larger impact if it was more visible in the school. The end result of this step were the results from the peer research on EBRBs.

##### 2.3.1.2 Step 1b: gaining insight into the system

In step 1b, the PAR groups developed CLDs for each of the EBRBs they focused on based on the results from their peer research. The research question underpinning the development of the CLDs was: what factors explain the unhealthy behaviour (specified for each EBRB) of 10–14-year-old adolescents in Amsterdam (East)? The co-researchers reported the factors which they identified from their peer research, whilst the facilitator then put it into the CLD using the software STICKE-2. During the process of developing the CLDs, the facilitator explained that the focus should be on those factors that people can change, thus excluding factors like the weather that are outside of their control. The co-researchers made connections between the factors with help from the facilitator until no further connections could be identified. Factors with no connections were subsequently removed from the CLD. Next, the PAR groups focused on identifying feedback loops and, when necessary, optimising the connections between the factors to complete the feedback loops. Furthermore, each CLD was verified by another PAR group as a member check or validation of the CLD. The PAR groups analysed all the CLDs from their school setting by checking the connections and identified feedback loops. Based on all the CLDs from their school setting, the PAR groups then proceeded to identify mechanisms, which were understood as overarching themes that influence EBRBs. The end result of this step were six CLDs, for each of the EBRBs and school settings (i.e., primary and secondary schools) and identified mechanisms.

#### 2.3.2 Step 2: programme outcomes and objectives

During this step, the PAR groups specified what needed to change in order to improve adolescents’ EBRBs based on the mechanisms from the previous step. The PAR groups determined which mechanisms needed to be addressed and subsequently which leverage points, using the ILF framework. An ILF table was completed for each mechanism based on the following questions: (1) what should change in our society/city/world to improve the lifestyle of adolescents? (2) What goal are we in the current project striving for? (3) What needs to be done in order to achieve that goal? (4) Who needs to be involved to accomplish these changes? The facilitators also encouraged the adolescents to formulate what needed to be done to disrupt the system, which enabled them to formulate leverage points. These ILF tables were used instead of matrices that are normally used in the IM protocol. The end result of this step were leverage points on the ILF levels that formulated goals to change the system that shapes adolescents’ EBRBs.

#### 2.3.3 Step 3: programme design

In this step, we developed a programme design for the actions, beginning with action ideas. We used two approaches to develop action ideas including (1) creative design sessions and (2) a co-creation process within PAR sessions. The creative design sessions included two-days of cooperation between co-researchers, academic researchers, municipality workers and important stakeholders from the target communities to develop action ideas. Within the co-creation process, the PAR groups developed ideas for their actions based on the leverage points they determined in the previous step. The facilitators encouraged the co-researchers to develop actions ideas for deeper ILF levels by using role-playing activities, whereby the co-researchers would select and play various people of influence. For example, we asked the co-researchers to imagine what they would do to improve the EBRBs of adolescents if they were the mayor of Amsterdam or a witch with magic powers. Both the impact and feasibility of the proposed action ideas were discussed by filling in an impact and feasibility matrix, which helped them to decide which actions to choose. We expected that some of the action ideas might either be too difficult or take too long for the co-researchers to develop and implement, and therefore explained that the co-researchers could choose to pass on these action ideas (or part thereof) to academic researchers within LIKE. Based on the expected impact and feasibility, as well as input from extant literature on effective potential strategies, the co-researchers chose several action ideas to develop further. Throughout this step the facilitators and co-researchers carefully checked if the chosen actions were in accordance with the leverage points identified in the previous step as well as if any unintended adverse effects were being overlooked.

#### 2.3.4 Step 4 and step 5: programme production and programme implementation plan

Steps 4 and 5 were combined because the actions were developed and implemented iteratively by the PAR groups. The PAR groups prepared the actions in detail, including by creating an implementation plan, and then implemented the actions. Preparing the action in detail included developing the structure of the programme, making a list of materials and ensuring that the action was ready for pilot testing. The co-researchers filled in a form to facilitate this process, which included the following questions: what do we need to know before we spend money and time on this action? What is the action? When will this action take place? Where will the action take place? How long will the action take? Who needs to support our action?

When developing the implementation plan with the PAR groups, we spent additional attention on the following questions: who do you need to carry out this action? What materials do you need? When should the action begin? How long should the action take? Do you think this action could also generate unintended adverse consequences? If not, then why not? If so, then how can we take this into account? Although these questions were often already discussed throughout the process of preparing the actions in detail, they were used as a final check to ensure that the co-researchers had properly thought these questions through. The end result of this step was a detailed plan for accomplishing the action adoption and implementation.

#### 2.3.5 Step 6: evaluation

In this step, the PAR groups conducted an evaluation on the implemented actions, such as the participation rate for the actions, participants’ satisfaction, target group reach and perceived impact on lifestyle behaviour. For example, the co-researchers could prepare surveys or interviews for the adolescents participating in the actions to determine both their satisfaction levels and the perceived impact on their lifestyle behaviour. The results of this evaluation were then used to adapt the actions (and their implementation) to improve the structural implementation of the actions. The overall evaluation of the impact and process of all the actions across the schools, which includes interviews with the involved stakeholders and schools, will be published in a separate article (39).

## 3 Results

Table 1 presents an overview of the number and content of the PAR sessions per research step.

**Table 1:** Overview of the number and content of the PAR sessions per research step.

Research steps	Number of sessions	Content PAR sessions
Mapping the system	Step 1a: 11–20 sessions in year one 13 to 16 sessions in year two^1^ Step 1b: 1–3 sessions for each Causal Loop Diagram	An academic researcher instructed the co-researchers on the principles and methodologies of conducting research The co-researchers conducted peer research (e.g., interviews, questionnaires) Creating Causal Loop Diagrams based on the results from the peer research
Programme outcomes and objectives	Step 2: 1–2 sessions	The co-researchers formulated programme objectives based on identified mechanisms Academic researchers filled in the Intervention Level Framework forms
Programme design	Step 3: 1–4 sessions	The academic researchers facilitated role-playing exercises to encourage the co-researchers to think beyond their own sphere of influence when developing action ideas Impact and feasibility matrix was used to help the co-researchers to choose actions based on the highest perceived impact and feasibility
Programme production and programme implementation plan	Steps 4 and 5: up to 18 sessions for each PAR group	The co-researchers worked in sub-groups to develop the selected actions in detail Structure of the action List of materials Ensuring action is ready for pilot testing The academic researchers facilitated filling in an action form to help the co-researchers think of the details (e.g., what do we need to know before we spend money and time on this action?) The implementation plan was written by the facilitator, based on input from the co-researchers through the following questions: Who do you need to carry out this action? What materials do you need? When should the action begin? How long should the action take? Do you think this action could also generate unintended adverse consequences? If not, why not? If so, how can we take this into account?
Action evaluation	Step 6: 0–3 sessions for each action, depending on whether the facilitator or co-researchers conducted this evaluation.	The co-researchers developed and administered questionnaires to evaluate actions In some instances the facilitator had to take over the evaluation of the actions due to time constraints or COVID-19

^1^Each PAR session lasted 45 min–2 h.

### 3.1 COVID-19

The PAR sessions were partly conducted during the COVID-19 pandemic. In a previous publication we described this in more detail (33). In the second and third years of the project, schools were closed multiple times due to the lockdowns. During the lockdowns, we continued our PAR sessions online amongst secondary school groups and either online or face-to-face outdoors with the primary school groups. We slightly adapted the form of the sessions, in order to make them attractive for the co-researchers in an online setting. At primary schools, the online sessions were not continued in year three during the second lockdown, due to concentration and motivation problems. As a result, the primary school PAR groups only conducted research step 1 and an evaluation session in year 2. In the secondary schools, the online PAR sessions went well and the sessions continued online during each of the subsequent lockdown periods. In year three, we continued with step 2 for the primary schools. For the secondary schools, we also started again in year 2 as new co-researchers were recruited for the PAR groups and we developed new actions with these groups.

### 3.2 Step 1: mapping the system

#### 3.2.1 Step 1a: gaining insight into energy balance-related behaviours

Table 2 presents an overview of the number of sessions used for this step for each PAR group. In summary, step 1a took between 11 and 20 sessions which each lasted 45 min–2 h, and took up the first 2 years of the project. In each new school year, we started with an almost completely new PAR group, which made it necessary to repeat some steps to help them both gain insight into their own behaviours and complete their own peer research. Additional sessions in the second year were necessary to complete the needs assessment for all EBRBs. Table 2 also presents the various methods that the co-researchers used to gain insight into their own behaviour. The co-researchers could suggest other methods than those proposed by the facilitators. For example, one group invited an expert on sleep behaviour, whilst another group suggested that they could draw their own sleep routine. This helped the co-researchers to better understand their sleep behaviour and conduct higher quality peer research by developing more specific research questions. After the capacity building session on basic research principles and methods, each PAR group developed research questions and choose their own peer research method. In the first year, the co-researchers expressed that gaining insight into their own behaviour was time consuming and, as such, boring. They instead wanted to start with conducting their peer research. Therefore, the facilitators scheduled less time for this step in the second year and with subsequent PAR groups.

**Table 2:** Overview of the number of sessions and methods used to gain insight into their own behaviour in each PAR group.

Step 1a	Number of sessions/total PAR sessions in that year	School year 1 (2018–2019)	Number of sessions/total PAR sessions in that year	School year 2 (2019–2020)
**Primary schools**				
School 1	11/12	Made a timeline of their physical activity during a weekday Conducted a neighbourhood safari	19/26	Discussed reasons for unhealthy behaviour
School 2	20/24	Made a timeline of their sleep behaviour during a weekday Discussed sleep routine with the PAR group	17/26	Consultation from a dietary behaviour expert
**Secondary schools**				
School 1	20/29	Made a timeline of their dietary behaviour during a weekday and weekend day Conducted a neighbourhood safari focusing on the food environment	13/20	Made a timeline of their dietary behaviour during a weekday Conducted a neighbourhood safari focusing on the food environment
School 2	14/28	Made a timeline of their physical activity, screen time, sleep behaviour and dietary behaviour during a weekday Invited a sleep expert for consultation	16/23	Made a timeline of their physical activity, screen time, sleep behaviour and dietary behaviour during a weekday Searched for information on the internet regarding these behaviours Discussed their sleep routine with each other

Step 1a: gaining insight into their own behaviours.

Almost all the data collection for the peer research took place at school during the PAR sessions amongst schoolmates from the last two grades of primary school and the first two grades of secondary school. Table 3 provides an overview of the peer research methods used by the co-researchers as well as the results for this step in each school year. In summary, the PAR groups conducted various quantitative and/or qualitative analyses of their data (e.g., in Excel). In the first school year, the facilitator and school board initiated a presentation of the preliminary results. The co-researchers prepared their presentations and decided on the format, e.g., a poster, movie clip or PowerPoint. Most co-researchers enjoyed making a poster because they could draw their results, including illustrations. The PAR groups presented their research either during an open day or at the creative design session in step 2 to increase awareness over adolescents’ current EBRBs. The facilitators planned to present the results to the school board in order to increase the visibility and impact of the co-researchers’ work. However, due to a lack of prioritisation from the board the co-researchers ended up presenting their results to the other sub-groups within the PAR group. In the second year, when the peer research was completed, the facilitator summarised the results for each behaviour in a long list of factors for each of the EBRBs as preparation for step 1b. Appendix step 1a provides an example of such a long list.

**Table 3:** Overview of the peer research methods and results of step 1a.

	School year 1 (2018–2019)		School year 2 (2019–2020)	
**Primary schools**				
School 1	Target EBRBs	Physical activity and sedentary behaviour	Target EBRBs	Physical activity and sedentary behaviour
	Methods	15 interviews regarding sports, gaming and unhealthy food products 11 questionnaires on the behaviours including drawings of the participants’ favourite sport	Methods	12 interviews regarding physical activity and sedentary behaviour 11 diaries in which adolescents logged their EBRBs for seven days
	Results	Due to time constraints this group had not analysed their peer research	Results	A list of perceived factors influencing adolescents’ own EBRBs
School 2	Target EBRBs	Sleep and dietary behaviour	Target EBRBs	Sleep and dietary behaviour
	Methods	26 interviews regarding sleep time, screen time and dietary behaviour before bedtime 26 questionnaires regarding behaviour before bedtime including screen time	Methods	19 interviews overarching on the behaviours including concentration at school 9 questionnaires on sleep, dietary and physical activity behaviour
	Results	A list of perceived factors influencing adolescents’ own EBRBs and a preliminary CLD	Results	A list of perceived factors influencing adolescents’ own EBRBs and a preliminary CLD
**Secondary schools**				
School 1	Target EBRBs	Dietary behaviour	Target EBRBs	Dietary behaviour
	Methods	72 questionnaires on dietary behaviour specifically during the school break	Methods	14 interviews regarding the school canteen and supermarket close to school
	Results	Dietary behaviour of adolescents in the first and second grade at school presented via poster presentations and a short movie clip	Results	A list of perceived factors influencing adolescents’ own EBRBs
School 2	Target EBRBs	Sleep behaviour and physical activity	Target EBRBs	Physical activity
	Methods	5 interviews regarding bedtime, screen time, physical activity and dietary behaviour before bedtime 1 focus group (*n* = 20) overarching on sleep behaviour 19 questionnaires on bedtime (rules), bedtime routine, screen time before bedtime	Methods	5 interviews on sport 34 questionnaires on sport and active/passive transport
	Results	Sleep behaviour of the first-grade adolescents at school presented via a poster presentation	Results	A list of perceived factors influencing adolescents’ own EBRBs

^Overview of the peer research methods used as well as the results from the research conducted by the PAR groups in the primary and secondary schools as part of step 1a.^

#### 3.2.2 Step 1b: gaining insight into the system

In the second year of the project, the PAR groups developed CLDs for each EBRB, which took approximately one to three sessions for each CLD (Appendix step 1b). As the creative design sessions were planned in the first year, we developed a preliminary CLD whilst the needs assessment was still ongoing. The CLDs were developed based on the long list of factors for each of the EBRBs established in step 1a. We experienced that the inclusion of all the factors in one CLD made the CLD hard to interpret. Therefore, the co-researchers first selected the 20 most important factors from the long list as a starting point for the CLD. The co-researchers could then easily choose the most important factors and draw connections between their prioritised factors. Here, the co-researchers needed encouragement from the facilitator to include additional factors required to complete the feedback loops. For example, to create the feedback loop ‘notifications ➔ staying up-to-date ➔ being curious ➔ notifications’ (Appendix step 1b), the facilitator discussed with the co-researchers why getting notifications led to picking up their phone multiple times before bedtime and kept asking follow-up questions until the feedback loop was complete. Verifying the CLDs from other PAR groups often resulted in small changes being made, such as removing or adding a factor. When discussions came up about making changes, the co-researchers then consulted a number of schoolmates, which often led to a clear decision on how to further improve their CLD.

In year two, not all CLDs were completed, therefore the PAR groups identified their focus of action based on the CLD(s) of their focus behaviour(s). Whereas in year three, the co-researchers determined their focus of action based on mechanisms across all focus behaviours. The PAR groups identified mechanisms by identifying overarching themes across the CLDs from all focus behaviours created in their school group. By motivating the PAR groups to think more broadly than the factors outlined within the CLDs, they were able to identify mechanisms. The facilitators used the exercise ‘five times why’ in which they kept asking the why question to encourage the co-researchers to identify overarching themes instead of single factors. One PAR group from the secondary school group identified the mechanism ‘bullying.’ They expressed that bullying led to being forced to engage in certain behaviours, such as joining their peers in going to the supermarket to buy unhealthy foods and drinks. Furthermore, they explained that being bullied induced stress, which, in turn, resulted in consuming unhealthy products and experiencing difficulties with falling asleep. A detailed description of the results of step 1, including the developed CLDs, can be found elsewhere (33).

### 3.3 Step 2: programme outcomes and objectives

Step 2 took one to two PAR sessions. To keep the PAR groups motivated, we already began developing actions during the needs assessment. The PAR groups decided on their focus of action when working on their poster (and other) presentations and preliminary CLDs. Next, the PAR groups decided which factors needed to be changed based on the feedback loops they had identified.

Although the facilitators developed an adolescent-friendly version of the ILF table, the co-researchers still perceived filling in the table as too difficult and time consuming. Furthermore, the co-researchers expressed their eagerness to develop actions by presenting action ideas as opposed to spending more time on filling out a ‘boring’ ILF table. Therefore, the facilitators decided to let the co-researchers formulate goals, based on the mechanisms from the previous step, for their action ideas that corresponded with leverage points. The facilitators encouraged the co-researchers to formulate what needed to be done to disrupt the system, which enabled them formulate leverage points. For example, for the mechanism ‘peer pressure’ co-researchers formulated the goal to help other adolescents to make their own choices despite peer pressure and providing them with information on how to deal with bullying.

The facilitators used these goals and information from discussions when formulating these goals to fill out the ILF tables themselves. For the primary schools, no ILF tables were filled in because the facilitator was short of time. The facilitators monitored the quality of the ILF tables through engaging in discussions with the research team, including the facilitators, TA and CD, and comparing them to ILF tables on similar topics which were made by the wider LIKE consortium. Appendix step 2 provides an example of a completed ILF table, based on the mechanisms ‘bullying’ and ‘peer pressure’. This ILF table was based on another ILF table that was previously drafted by LIKE consortium members, addressing the related mechanism ‘social norms and acting cool’. The facilitator used this table as a basis and made changes based on the goals that the co-researchers formulated, which are highlighted in yellow. One example of a leverage point that was added by the facilitator based on the co-researcher formulated goal is ‘Actors–including adolescents themselves–are aware of the prevailing norms regarding unhealthy lifestyles and the peer pressure that is associated with this.’

### 3.4 Step 3: programme design

**Figure 2:**
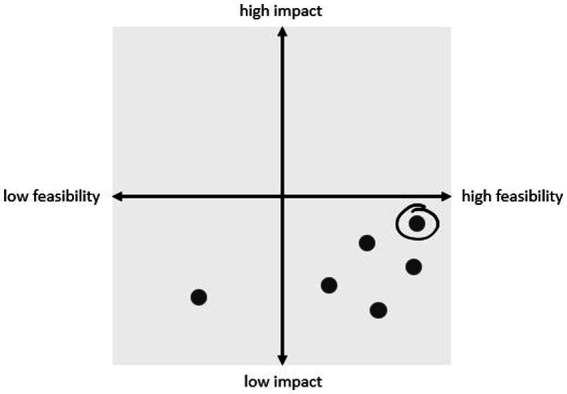
Example of impact and feasibility matrix with all action ideas rated for impact and feasibility.

Step 3 took one to four PAR sessions. In the first project year this step was facilitated by a two-day creative design workshop: two PAR groups (one primary and one secondary school group) joined the workshop along with academic researchers, municipality workers and other relevant stakeholders (e.g., community organisations). During this creative design workshop, the PAR groups designed pilot actions with feedback rounds of other workshop participants. Starting in project year 3, the co-researchers identified leverage points and developed actions targeting these leverage points. To encourage the co-researchers to think beyond both their own sphere of influence and potential challenges related with feasibility, we introduced role-playing exercises. Appendix step 3 provides an example of a list of the developed action ideas. The impact and feasibility matrix (Figure 2) proved to be a useful instrument for helping the co-researchers to choose actions which they expected would generate the most impact and which were most feasible. The facilitator provided advice based on extant scientific evidence, by, for example, explaining that providing knowledge alone is insufficient for behaviour change. The co-researchers also gathered feedback from one another, which further improved the action ideas. For example, for the mechanism ‘parents are responsible for adolescents’ sleep behaviour’, one PAR group combined the three action ideas (1) more rules from parents, (2) explain to parents why adolescents often do not sleep well, and (3) teaching parents where adolescents hide sweets in their room, into the idea of organising an interactive workshop with parents about rules regarding bedtime, screen time, and so on. The co-researchers chose this as one of the actions they wanted to further develop and drafted a first detailed outline of the action design. For example, the workshop should include discussion and reaching a consensus between the parent and child before implementing a new rule, as adolescents would then be more inclined to follow this rule. The facilitator encouraged the co-researchers to think about any potential unintended consequences of the actions by discussing their concerns about their action design. For example, when brainstorming about a healthy canteen week, the co-researchers mentioned that the canteen could display the calories within each product. However, when asked to think about the adverse effects of this practise, the co-researchers noted that they knew of some adolescents who already focus too much on calories and they did not want to exacerbate this. To ensure the highest possible impact and feasibility, each action design was also discussed within the research team and checked with the CLD and leverage points from the ILF tables. The facilitator discussed any potential concerns regarding each action design with the co-researchers, before then proceeding to work together to improve each action design.

### 3.5 Step 4 and step 5: programme production and programme implementation plan

Steps 4 and 5 took up to 18 sessions for each PAR group per year. As part of these two steps, the chosen actions were developed in greater detail, which included producing an implementation plan. A detailed overview of all developed and implemented actions will be part of our process evaluation. As each PAR group developed multiple actions, the co-researchers worked in sub-groups on their actions. In order to make the actions more concrete and specific, the facilitators adapted the form into an adolescent-friendly version as the form contained difficult words (Appendix step 4 and 5). Although the co-researchers understood the improved version of the form, they still did not enjoy filling it out, because they experienced it as boring. Therefore, the facilitators asked them the questions listed on the form during the sessions themselves. Some actions were discontinued during this step as the PAR groups concluded that these actions would probably not improve the EBRBs of adolescents. For example, one PAR group tried to develop a video game which educated adolescents about healthy sleeping behaviour by obliging that their avatar had to sleep at night to regain their energy. If they turned the game off around bedtime and throughout the night, then the avatar would also go to sleep and the player would gain extra points, which, in turn, would stimulate healthy sleeping behaviour. However, whilst developing prototypes of the game the co-researchers realised that they would still find ways to continue their screen time on different devices or apps. Therefore, the game would not improve their sleep behaviour and the action was discontinued.

In the event that it was needed, the facilitators would provide the co-researchers with more knowledge to help them develop their actions in greater detail. For example, when the co-researchers wanted to develop an action directed at supermarkets, the facilitators organised a trip to the supermarket to help the co-researchers envisage their action. One PAR group wanted to use nudging in the school canteen to persuade adolescents to make healthier choices. Thus, the facilitator invited a nudging expert to provide the co-researchers with knowledge on implementing different types of nudging, and assisted them in developing their action.

What worked well for the PAR groups was having them simultaneously develop their implementation plan whilst preparing the actions in more detail. The co-researchers did not enjoy writing implementation plans as it was perceived as being boring and time-consuming. Therefore, the facilitators took over the writing of these implementation plans. When possible, the facilitators paired the PAR groups together with relevant stakeholders to develop actions. The stakeholders provided advice, funding, materials, a location, etc. For example, one PAR group organised a health festival together with municipality workers within an existing health festival, which included a play by the co-researchers designed to inform parents about adolescents’ sleeping behaviour. A ‘comic bad example’ of parenting regarding rules on sleep behaviour was presented followed by a good example of parenting.

### 3.6 Step 6: evaluation

The co-researchers evaluated the implemented actions on the basis of their perceived effect and impact upon the participants. For example, regarding the action ‘healthy canteen week,’ the co-researchers developed an online questionnaire that was completed by 115 of their peers (Appendix step 6). The co-researchers developed this questionnaire together with the facilitator, and when needed also received feedback from the research team. It included questions such as: what do you think about the canteen being healthy this week? What would you think if some of the products were always healthy? Did you go to the canteen this week? The co-researchers presented the results of this evaluation to members of the school board who expressed their desire for a healthier canteen the following year.

In some instances the facilitator had to take over the evaluation of the actions due to time constraints or COVID-19; for example, some actions were implemented when the PAR group were already finished. The co-researchers did not always enjoy developing an evaluation plan, because they had already spent a lot of time developing and implementing the action. However, when they were presented with the results from their own questionnaire, they generally got really excited and gained a better overview of the impact their action had.

## 4 Discussion

This paper describes how we applied PAR and system dynamics to each step of the IM protocol and how these steps were implemented in real life to develop, implement and evaluate actions to stimulate healthy EBRBs together with adolescents in an underserved neighbourhood in Amsterdam, the Netherlands. By using this novel approach, we developed actions that better fit the needs and wishes of adolescents and target different levels of the system (i.e., paradigm, system structure, feedback and delays, and structural elements), which, in turn, might positively impact upon overweight and obesity.

Through extensive peer research, the co-researchers gained insight into adolescents’ EBRBs, the most important factors influencing EBRBs as well as the connections between these factors. Gaining insight into these lived experiences helped the co-researchers to develop the CLDs and increase their understanding of the wider system and its influence on adolescents’ EBRBs. From the CLDs, the co-researchers then identified overarching mechanisms across all EBRBs, thereby focusing on feedback loops instead of single factors. This, in turn, led to goals and subsequently actions that were based on overarching mechanisms. Based on these overarching themes the co-researchers developed action ideas that intervened at deeper levels of the system. The co-researchers also drew upon their own lived experience in the action development, by explaining to the facilitator why they thought an action would work or not and what improvements were needed to impact upon adolescents’ EBRBs. This led to actions with potentially greater impact as it better fitted their needs, preferences and lived experience, which, in turn, increases the potential impact of the actions (40). Skilled facilitation is key to conduct this process with adolescent co-researchers (41). The facilitators should focus on the collaboration between the adolescents and adults within the school setting, this involves fostering trust and respect, creating meaningful roles, capacity-building of the adolescents and an optimal group size (42).

The PAR groups developed actions with leverage points for four out of the five system levels (34). For example, an action with a leverage point at the “paradigm level” was a podcast with episodes on the unhealthy food environment and various aspects of well-being, such as bullying and peer pressure. An action with a leverage point at the “system structure level” action involved the PAR group presenting their ideas about the creation of a healthier neighbourhood to a local administrative. An example of an action with a leverage point at the “feedback and delay level” was an interactive evening at school for parents about adolescents’ sleeping behaviour. An example of an action with a leverage point at the “structural elements level” was a healthy lunch assignment the PAR group set up during COVID-19 lockdown, where adolescents prepared and ate three healthy lunches during 1 week as part of their homework. These actions with leverage points at different levels of the system increase the opportunity for system change, which requires actions on the deeper levels of the system such as the paradigm level (43).

The IM protocol provided the structure for the development, implementation and evaluation of actions as well as ensuring that the actions were based on both empirical evidence and theory (29). For example, the IM protocol provided guidance in step three, programme design, where the facilitators presented potential effective action strategies based on extant literature as inspiration for the co-researchers. The facilitators also explained to the co-researchers when certain action strategies would potentially be insufficient for behaviour change, such as in the case of actions that were only based on education. Previous research combining IM and PAR with children also showed that including IM provided the necessary structure to take a step back and reflect on the children’s ideas, include guidance from current evidence, and maintain the enthusiasm of the children to develop and implement actions (27).

During the process, we experienced that the successful application of PAR combined with system dynamics and IM was highly dependent on the skills of the facilitators and both their knowledge of and experience with PAR, system dynamics and the IM protocol. Advanced facilitation skills are needed and can be acquired through a facilitation course and by joining more experienced PAR facilitators as co-facilitators in their sessions (41). Advanced facilitation skills help to foster active and equal collaboration with co-researchers by creating and maintaining a safe, functional and positive atmosphere, which, in turn, ensures ownership and shared decision-making (41). Prior experience with facilitating PAR sessions helped the facilitators to translate system dynamics and IM into exercises that were understandable, fun and interesting for the adolescent co-researchers. Moreover, facilitators should be guided by researchers with expertise on system dynamics, PAR and IM to support appropriate application of these approaches.

The main challenge with our approach was to strike a good balance between theory and practise during each step of the IM process, whilst, simultaneously, keeping the process interesting and active enough for the adolescent co-researchers. The facilitators needed to continuously assess if the planned exercises and sessions were successfully conducted by the co-researchers or whether it was necessary to adapt the approaches, by, for example, having more boring tasks be conducted by the wider research team. The wider research team included researchers with expertise on PAR, system dynamics and IM. However, as the practical application of system dynamics is still relatively new, applying system dynamics in PAR with adolescents required some ingenuity and trial and error. For instance, we did not succeed in translating the theoretical step of filling out the ILF tables into an attractive exercise for the co-researchers, who perceived this to be too time consuming and boring. Future research might experiment with using more practical frameworks to identify leverage points with co-researchers; one useful approach might be the recently developed Action Scales Model, which was developed to help practitioners and policymakers to conceptualise, identify and appraise actions within a complex adaptive system (44). Previous research has also shown that multiple facilitators are recommended for each PAR group, because they can give more individual guidance, manage the co-researchers better and get more work done in one session (41). Indeed, working in sub-groups of 2–3 adolescents, with each group being supported by one facilitator, helped to guide the co-researchers through the more theoretically oriented tasks in the process.

### 4.1 Strengths and limitations

One strength of this study is the detailed and critical description of the research process provided therein. There are few publications that provide this, and thus by doing so we hope to inspire other researchers to publish an extensive description of their research process. A further strength is the extensive equal collaboration with the adolescent co-researchers, in comparison with previous research conducting a systems approach such as CO-CREATE. In CO-CREATE, 20 groups of adolescents participated in a one-off 1.5 h session to develop a CLD, with the CLDs then being combined into one CLD by the researchers (20). Therefore, the researchers only collaborated with the adolescents once, albeit they did develop 20 CLDs. One limitation of this study is that we did not succeed in making each step of the process accessible for the adolescents; for example, filling out the ILF tables proved not to be an attractive exercise for the co-researchers. This is important because as researchers we should invest more time and energy in conducting inclusive research, as it is our duty to develop accessible and attractive methods to better include our target group. Furthermore, the LIKE consortium cooperated with various community partners, which helped to include stakeholders within both the development and implementation of the actions. Despite the outbreak of the COVID-19 pandemic, the facilitators managed to keep in contact with the co-researchers and continued with the PAR groups in ways that were possible at that time. Despite this, we could not avoid a delay in the process, some steps could not be conducted as planned, and at times the facilitators had to conduct tasks without the co-researchers.

## 5 Conclusion

We experienced that combining IM, PAR and system dynamics worked well in developing actions that meet adolescents’ needs and target different levels of the system. PAR ensured that the actions better fit the needs and lived experience of adolescents living in an underserved neighbourhood in Amsterdam, which is especially valuable for this population as they are underrepresented in most interventions and research. System dynamics promoted taking into account the wider system, including the search for feedback loops and leverage points that influence behaviours, which, in turn, result in actions targeting different levels of the system. The IM protocol ensured a structured process and that the work built on existing theory and empirical evidence. The success factors of such an approach include skilled facilitators focused on youth-adult collaboration, and guidance by a research team with adequate knowledge of and experience with PAR, system dynamics and IM. The main challenge pertained to the practical application of system dynamics as well as how to successfully collaborate with adolescent co-researchers. Future research could explore how certain aspects of the system dynamics approach could be adapted to better fit the participatory approach with adolescent co-researchers. In this respect, this study can serve as an example to other studies in how to develop actions using IM, PAR and system dynamics.

## Data availability statement

The raw data supporting the conclusions of this article will be made available by the authors, without undue reservation.

## Ethics statement

The studies involving humans were approved by the Medical Ethical Committee of the VU University. The studies were conducted in accordance with the local legislation and institutional requirements. Written informed consent for participation in this study was provided by the participants’ legal guardians/next of kin and the adolescents themselves.

## Author contributions

HE: Writing – original draft, Writing – review & editing. TA: Writing – review & editing, Supervision. CD: Writing – review & editing, Supervision. AP: Writing – review & editing. KS: Writing – review & editing. WW: Writing – review & editing. SK: Writing – review & editing, Supervision. MC: Writing – review & editing, Supervision.
