# Supplementary material for: Applying systems thinking in youth-centred participatory action research for health promotion in an underserved neighbourhood
Source: Front Public Health. 2024 Jun 3;12:1272663. doi: 10.3389/fpubh.2024.1272663 (PMC11180748; doi:10.3389/fpubh.2024.1272663)
Supplement: Supplementary file 1 [file Table_1.DOCX]

Appendix

# Appendix: Step 1a

Long list of factors from the co-researchers’ peer research that influence the sleep behaviour of secondary school adolescents (first two grades)

*Please note that it is from this list that the co-researchers subsequently chose the twenty most important factors as the starting point for their CLDs.*

| - Going to bed late - Sleep routine (what you do before you go to sleep)   - Reading   - Using your phone before bedtime   - Gaming   - Listening to music (quiet/soft)   - Using sleep medication - Sleep routine of the rest of the family - What you have to do the next day/important day   - Appointments the next day   - Sporting competition   - Birthday (either your own or that of a family member)   - Outings - Lying awake AND falling asleep   - Lots of energy   - Contemplating   - Noise   - Light outside   - Lamp on   - Not looking forward to the next day   - Nightmare   - Temperature in the bedroom     - Feeling cold     - Feeling warm in the bedroom - Exercising in the evening - Dinner time - Parents’ rules - Boring parents (more rules/or only allowed to do certain activities with parents) - Done a lot that day/used a lot of energy   - Activities     - Cycling     - Sports - Was it a school day - Long day - Watching ASMR (autonomous sensory meridian response) videos - Not feeling tired - Eating before bedtime - Using phone at night/before sleeping   - Checking school grades   - Setting alarm clock   - Social media such as Instagram and WhatsApp   - Curious about what others say and think   - Notifications   - Netflix and YouTube     - auto play   - When I have nothing to do   - There is nothing to do on your phone - Gaming   - Tablet   - Watching TV   - Waiting for the series   - Being at home   - Coming home after school   - Not feeling like playing with brother or sister   - Friends ask me to come play with them   - I want to get better at gaming   - I want to win   - Gaming is fun   - If you broke your gaming equipment - Weekends   - Meeting up with friends   - Sleeping in - Nothing to do the next day - Playing outside - Chilling outside with friends |
| --- |

# Appendix: Step 1b

CLD on the sleep behaviour of secondary school adolescents


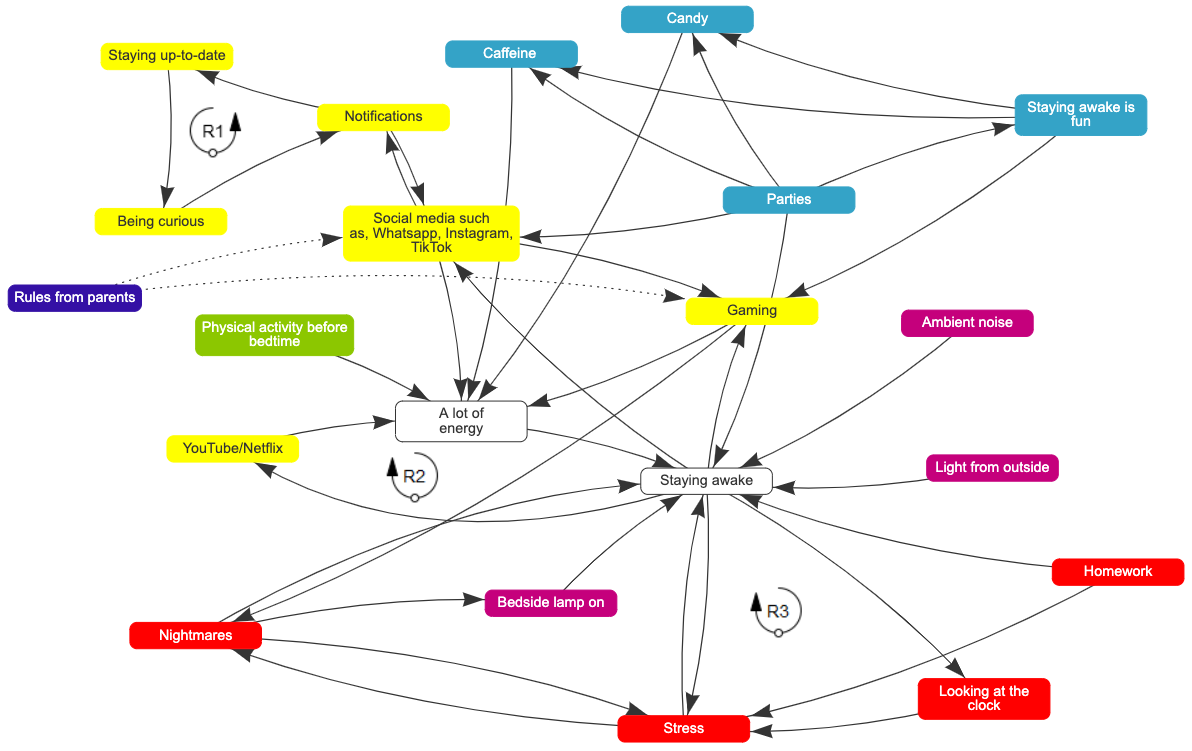


Similar colours represent related factors

# Appendix: Step 2

This appendix shows a completed ILF table for the mechanisms ‘bullying’ and ‘peer pressure’. This ILF table was based on another ILF table that was previously drafted by LIKE consortium members, addressing the related mechanism ‘social norms and acting cool’. The facilitator made additions to this ILF table based on the information from the co-researchers, these are highlighted in yellow.

Table 3. Completed ILF table for the mechanisms ‘bullying’ and ‘peer pressure’.

| **Five leverage points according to the Intervention Level Framework** | | | | |
| --- | --- | --- | --- | --- |
| Paradigm | System goal | System structure | Feedback & delay | Structural elements |
| **What are the leverage points to change mechanisms at each of the five ILF levels, in order to create a system that produces healthy behaviour** | | | | |
| *Which paradigm can contribute to adolescents exhibiting less unhealthy behaviour because they want to belong to the group? Note either an existing paradigm (that needs to be reinforced) or a new paradigm (requires a paradigm shift).* | *What goals should we set to achieve this paradigm shift?* | *What changes need to take place in the structure of the relevant system to achieve these goals?* | *In what ways can monitoring and feedback positively strengthen the mechanism, and how could we possibly build in monitoring/evaluation activities for this?* | *What elements in the system can you address with actions that support shifting the paradigm?* |
| New paradigm:  - As an adolescent you also want to be unique, and it is cool to regularly make your own choices in terms of your own lifestyle behaviours (own identity).  - Adolescents need to be protected from the pressures of social norms. | Actors – including adolescents themselves – are aware of the prevailing norms regarding unhealthy lifestyles and the peer pressure that is associated with this.  Actors in the system – e.g. adolescents themselves, parents, teachers, influencers, marketing industry – stimulate adolescents in making their own, unique choices aimed at a healthy lifestyle (i.e. healthy alternatives), in order to be autonomous.  Actors in the system – parents, teachers, influencers, marketing industry – help adolescents to come up with attractive, healthy alternatives.  In adolescents’ social environment, it is safe to talk to each other about a healthy lifestyle.  There is a clear policy (regulation) on supporting adolescents in terms of having a healthy lifestyle, at home, school, in the neighbourhood/city and online.  There is a clear policy regarding protecting adolescents from the pressures of social norms – at home, school, in the neighbourhood/city (e.g. in the supermarket) and online (e.g. rules regarding online screen behaviour, buying/eating unhealthy products during breaks and after school as a group activity).  Actors in the system – e.g. adolescents themselves, parents, teachers, influencers, marketing industry – set good examples regarding healthy lifestyles. | There is good communication between adolescents, parents, teachers, focused on healthy lifestyles (unambiguous message).  There is good collaboration between the government and actors (especially industry, local entrepreneurs, influencers) in the system that influence adolescents with respect to healthy lifestyles. | Actors talk to each other about their healthy lifestyle behaviour and adapt their behaviour accordingly.  Actors (especially industry, local entrepreneurs, influencers) are monitored as to whether they implement the policy on promoting healthy lifestyles.  Identifying barriers and facilitators for adolescents to make their own healthy lifestyle choices.  Identifying barriers and facilitators for adolescents to make their own healthy lifestyle choices.  Let adolescents experience that they will not be bullied, or be kicked out of the group, if they have a healthy lifestyle.  Adolescents experience a new group feeling when they choose a healthy alternative together with the group.  Letting adolescents experience that by making their own choices, they have more influence on their own lives (autonomy)  Actors are monitored to see whether they implement the policy on protecting adolescents from the pressures of social norms.  Actors – including adolescents – experience that their own healthy example has a positive influence upon other actors (including adolescents) in the system. | The school curriculum 1) supports adolescents’ own healthy lifestyle choices and prevents bullying behaviour; 2) protects adolescents from the pressures of social norms.  Parents are supported in 1) encouraging their children to make their own healthy lifestyle choices; 2) establishing and enforcing rules to protect adolescents from the pressures of social norms.  Entrepreneurs and influencers are supported in 1) how to communicate a healthy message to adolescents; 2) drafting and complying with policies to protect adolescents from the pressures of social norms. |
| **What actions can be taken to change this mechanism (e.g. define this in terms of function, and formulate the function so that it is SMART)** | | | | |
| Changing the normal behaviour from unhealthy to healthy (similar to “but I don’t smoke”).  Giving the responsibility back to the people who spread the message that unhealthy behaviour is “cool” (influencers, marketing) | Teach adolescents to regularly make their own, unique choices (e.g. doing sports instead of gaming) and to see this as being cool (making adolescents resilient).  Help adolescents to think of healthy alternatives and let them experience that that these are tasty and fun.  Develop policies – in the home, supermarket, neighbourhood – to protect adolescents against the pressures of social norms. | Actors (parents, schools, entrepreneurs) ensure a safe environment at school, home, and the sports club where people hold each other accountable for healthy and unhealthy behaviour.  Actors (parents, schools, entrepreneurs, influencers) make arrangements with each other to prevent ‘unequal competition’. | Message from influencers/role models about evaluating each other (cf. 'media code').  Adolescents share and learn from their experiences (barriers, facilitators) regarding implementing healthy lifestyles.  Actors – adolescents, parents, teachers, entrepreneurs, influencers – share their experiences (barriers, facilitators) on developing and enforcing rules designed to protect adolescents against the pressure of social norms.  Adolescents and experts confront each other about their health behaviour and adjust their behaviour accordingly. | Awareness about the importance of making one's own choices in adolescents’ immediate environment (home, school).  Activities to help adolescents make their own choices aimed at a healthy lifestyle (enhancing their skills and confidence).  Role models (influencers, peer educators) who make their own choices in healthy behaviour and share their experiences with adolescents.  Provide in-service training and support to entrepreneurs on how to communicate positive lifestyle norms.  Raise awareness amongst actors (parents, teachers, entrepreneurs, influencers) about the importance of protecting adolescents from the pressures of social norms.  Provide support to actors (parents, teachers, entrepreneurs, influencers) in creating and enforcing rules aimed at protecting adolescents from the pressures of social norms. |

# Appendix: Step 3

All action ideas to improve healthy dietary behaviour during school time in secondary school:

- Adolescents are not allowed to go to the supermarket anymore during the school break
- Healthy products are more readily available (on discount) than unhealthy products in the school canteen
- Unhealthy products are made more expensive in the canteen (and the supermarket)
- Healthy products are more visible in the school canteen compared to unhealthy products (easy to see/nudging towards healthy dietary behaviour)
- Existing products in the canteen are healthier than before, such as, for example, making Paninis on brown bread instead of white
- Healthy products are made cheaper in the canteen and the supermarket
- Adolescents put healthy products in their peer group’s shopping baskets in the supermarket.

This list of actions ultimately culminated in a healthy canteen week in which only healthy products were sold during that week. Adolescents also received some free healthy lunches in that week to help promote healthy canteen week. Furthermore, due to the COVID-19 lockdown, the co-researchers also developed a healthy lunch assignment, where adolescents had to make three healthy lunches at home during the course of one week. The adolescents had to make TikTok videos of their meals and show how to prepare them, and then hand this in.

# Appendix: Step 4 and 5

Form with questions to help develop the intervention production and implementation plan.

| **Research question** | | **Measuring:** | **Form:** |
| --- | --- | --- | --- |
| What should we be sure of before we spend a lot of time and money on it? | | % users, how often, how long? | What will you do? What will other adolescents notice? |
| **Details:** | **What do you need:** | **Learn:** | **Consequences:** |
| What, where, when, for how long? | Money, materials, commitment | How can we learn from our idea? | What is needed to continue with the actions?  When will you stop the actions? |
| **Sponsors:** |  |  |  |
| Who should support this idea? |  |  |  |

# Appendix: Step 6

Table 4. Evaluation of the healthy canteen week

| **Evaluation on the healthy canteen week** | | | | | |
| --- | --- | --- | --- | --- | --- |
| Perception of how the adolescents felt about the healthy school canteen (n=115) | Not nice  12.2% | I do not care  19.1 % | Okay/fine  10.4% | Good/fun  57.4% | Super cool  0.9% |
| Whether the adolescents had tasted something they had not tasted before (n=83) | Yes  19.3% | No  80.7% |  |  |  |
|  | 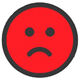 | 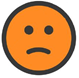 | 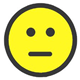 | 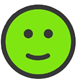 | 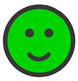 |
| Whether the adolescents liked what they had never tried before | 6.3% | 12.5% | 18.8% | 37.5% | 25% |
| Perception of the canteen staying healthy in the future (n=115) | 1.7% | 11.3% | 38.3% | 38.3% | 10.4% |
| Perception of the taste of the canteen food during healthy canteen week (n=77) | 0% | 3.9% | 20.8% | 48.0% | 27.3% |
